# Cost-effectiveness of iGlarLixi vs. IDegAsp in individuals with type 2 diabetes: a BRAVO model-based evaluation

**DOI:** 10.3389/fpubh.2026.1787562

**Published:** 2026-05-26

**Authors:** Jiali Qin, Man Tang, Xiaomei Wang, Lizheng Shi, Hui Shao, Vivian Fonseca, Xiaoyan You, Xianying Wang

**Affiliations:** 1 College of Pharmacy, Hebei Medical University, Shijiazhuang, Hebei, China; 2 Department of Pharmacy, Third Hospital of Hebei Medical University, Shijiazhuang, Hebei, China; 3 Department of Health Policy and Management, Celia Scott Weatherhead School of Public Health and Tropical Medicine, Tulane University, New Orleans, LA, United States; 4 Hubert Department of Global Health, Rollins School of Public Health, Emory University, Atlanta, GA, United States; 5 School of Medicine, Tulane University, New Orleans, LA, United States

**Keywords:** BRAVO model, cost-effectiveness analysis, IDegAsp, IGlarLixi, type 2 diabetes

## Abstract

**Background:**

This study aimed to evaluate the long-term cost-effectiveness of insulin glargine/lixisenatide injection (iGlarLixi) vs. insulin degludec/insulin aspart (IDegAsp) in individuals with poorly controlled type 2 diabetes in China.

**Methods:**

This study employed the Building, Relating, Assessing, and Validating Outcomes (BRAVO) diabetes model to simulate 20-year clinical and economic outcomes from the perspective of the Chinese healthcare system. Baseline cohort characteristics and treatment effects were derived from the Soli-D clinical trial (NCT05413369). Drug prices for iGlarLixi and IDegAsp were sourced from Yaozhi Complication-related costs and utility values were obtained from literature. The primary outcome was the incremental cost-effectiveness ratio. The willingness-to-pay threshold was defined as three times China's per capita gross domestic product ($13,448 in 2024) per Quality Adjusted Life Years (QALY). Both costs and utilities were discounted at 5% annual rate. One-way sensitivity, scenario, and probabilistic sensitivity analyses were conducted to assess the robustness of the findings.

**Results:**

Compared with IDegAsp, iGlarLixi treatment resulted in an additional gain of 0.03 QALYs with a saving of $301.38 (dominant), indicating a favorable economic outcome toward iGlarLixi over IDegAsp. Sensitivity analyses confirmed the robustness of the results. Within the threshold of $40,344/QALY, the probability of iGlarLixi being cost-effective compared with IDegAsp was 83%.

**Conclusions:**

In individuals with type 2 diabetes inadequately controlled by oral antihyperglycemic agents, treatment with iGlarLixi is associated with superior long-term clinical outcomes and lower healthcare costs compared to IDegAsp in the Chinese healthcare setting.

## 1 Introduction

The global burden of diabetes mellitus continues to escalate, with both incidence and prevalence rising at an alarming rate. According to projections from the International Diabetes Federation (IDF), the number of individuals affected by diabetes is expected to reach approximately 783.2 million by 2045, of which an estimated 90% will have type 2 diabetes (T2D) (1, 2). In China, the economic impact of diabetes is particularly pronounced: in 2021 alone, diabetes-related healthcare expenditures totaled USD 165.3 billion, ranking second globally after the United States (3). This substantial financial burden highlights the need for pharmacoeconomic evaluations to help choose therapies that are not only clinically effective and safe but also cost-efficient within the healthcare system.

Current international and Chinese clinical guidelines advocate for an individualized approach to glycemic control in patients with T2D, with treatment selection guided by patient comorbidities, cardiovascular risk, and weight management goals (4, 5). Pharmacologic therapy should generally be initiated promptly after diagnosis and used in conjunction with lifestyle modification to avoid therapeutic inertia and facilitate early achievement of glycemic targets (5). For individuals without established cardiovascular disease, heart failure, or chronic kidney disease, metformin monotherapy remains a commonly used initial treatment option (5). In contrast, for those with established or high risk of atherosclerotic cardiovascular disease, heart failure, or chronic kidney disease, glucose-lowering agents with proven cardiovascular or renal benefits—such as glucagon-like peptide-1 receptor agonists (GLP-1 RAs) or sodium–glucose cotransporter 2 (SGLT-2) inhibitors—are recommended irrespective of A1C levels (5). For individuals requiring treatment intensification, in the absence of insulin deficiency, GLP-1 RAs or dual glucose-dependent insulinotropic polypeptide (GIP)/GLP-1 RAs are preferred due to their lower risk of hypoglycemia and favorable effects on body weight. If insulin therapy is required, combination therapy with a GLP-1 RA is recommended to enhance glycemic control and improve metabolic outcomes (5).

Insulin glargine/lixisenatide (iGlarLixi) is a fixed-ratio combination of BI and GLP-1 RA that delivers complementary glycemic control—insulin glargine provides sustained 24-h basal glucose regulation, while lixisenatide targets postprandial glucose excursions. This dual mechanism may reduce the risk of adverse effects commonly associated with monotherapy. The efficacy and safety of iGlarLixi have been well extensively evaluated in diverse populations (6). Results from the global LixiLan clinical trials demonstrated that iGlarLixi improves fasting plasma glucose compared with BI monotherapy, while maintaining a comparable or lower risk of hypoglycemia (7). These studies demonstrated that iGlarLixi improves fasting plasma glucose more effectively than BI alone, while maintaining a similar or lower risk of hypoglycemia, and induces fewer gastrointestinal adverse events compared to GLP-1 RA monotherapy (6). In recognition of its clinical utility, iGlarLixi was included in China's National Reimbursement Drug List (NRDL) in 2023.

Similarly, insulin degludec/insulin aspart (IDegAsp) is a fixed-ratio formulation of 70% long-acting insulin degludec and 30% rapid-acting insulin aspart, providing comprehensive control of fasting and postprandial glucose (8). Administered once or twice daily via prefilled pen devices, it supports patient adherence. International and Chinese diabetes guidelines recommend IDegAsp for patients needing both basal and prandial glucose control or whose glycemic targets are not met with basal insulin alone (4). IDegAsp was approved in China in 2019 and added to the NRDL in 2020.

Despite the clinical availability and reimbursement coverage of both therapies, direct pharmacoeconomic evidence comparing iGlarLixi and IDegAsp in the Chinese healthcare context remains limited. Given that both are fixed-ratio combination therapies designed to simplify regimens, improve adherence, and provide comprehensive glycemic control, and that they share similar clinical indications and reimbursement status, a comparative cost-effectiveness evaluation is warranted. Therefore, the present study assesses the long-term cost-effectiveness of iGlarLixi vs. IDegAsp among adults with poorly controlled T2D in China, integrating data from clinical trials to inform diabetes management and healthcare resource allocation.

## 2 Methods

### 2.1 Modeling approach

The Building, Relating, Assessing, and Validating Outcomes (BRAVO) of Diabetes Model was used to assess cost-effectiveness of iGlarLixi vs. IDegAsp. The BRAVO model is a widely used and validated diabetes simulation model that utilizes a series of discrete intercorrelated risk equations to estimate long-term clinical outcomes (9–11). These include diabetes-related complications, hypoglycemia, mortality, and the progression of modifiable risk factors (9). The model has undergone extensive calibration and validation using data from multiple international clinical trials (12). Notably, it was previously applied in a 2022 study to simulate 5-year complication rates of iGlarLixi compared to Standard of Care, providing a solid foundation for its application in this study (13).

Clinical data were obtained from the Soli-D trial (14), a 24-week, randomized, active-controlled, open-label, parallel-group, multicenter phase 3 study. This trial compared the efficacy and safety of iGlarLixi vs. IDegAsp in Chinese adults with T2D who were taking metformin with or without one additional OAD. The trial results showed that HbA1c levels for both iGlarLixi and IDegAsp stabilized between weeks 18 and 24, indicating that a stable treatment effect had been achieved by week 24. Therefore, the treatment effect observed at week 24 was considered to represent the annual treatment effect, reasonably reflecting the average long-term treatment response. In accordance with the structural assumptions of the BRAVO model, treatment effects were assumed to persist for 5 years, consistent with the time horizon commonly applied in long-term pharmacoeconomic evaluations for diabetes (11). Specifically, Treatment effects were modeled as arm-specific differences in time-varying risk factors over the first 5 annual cycles. Thereafter, biomarker progression followed the BRAVO natural-history equations. Updated risk-factor values were propagated through the BRAVO events and mortality modules to estimate long-term complications, survival, costs, and QALYs. All costs, clinical outcomes, and utility values were discounted at an annual rate of 5%, consistent with the 2020 China Pharmacoeconomic Evaluation Guidelines (15).

### 2.2 Study population

The study population was drawn from the Soli-D trial (14), which enrolled 582 adult participants with T2D who exhibited inadequate glycemic control on OADs. Inadequate control was defined by HbA1c thresholds that varied depending on prior therapy: participants previously treated with metformin alone or metformin in combination with an SGLT-2i were required to have an HbA1c between 7.5 and 11.0%, while those treated with metformin and a second OAD (excluding SGLT-2i) were required to have an HbA1c between 7.0 and 10.0%. The inclusion criteria for this analysis followed the specifications outlined in the original clinical trial protocol.

Model inputs included demographic and clinical characteristics such as age, gender, diabetes duration, smoking status, and baseline clinical biomarkers (e.g., HbA1c, SBP, LDL, and BMI), along with treatment effect data (Table 1). Incidence rates of grades 1–3 hypoglycemia were also recorded (Table 1). These data were incorporated into the BRAVO model to simulate disease progression and treatment outcomes.

**Table 1:** Baseline characteristics of the population and model input Demographic and clinical characteristics of Chinese individuals with poorly controlled T2D included in the BRAVO model. Data include age, sex, duration of diabetes, HbA1c, and other relevant parameters used for model simulations.

Parameters		iGlarLixi	IDegAsp	Mean	Source
Individuals characteristic					
Age, (years)		56.30 (10.20)	57.50 (9.90)	56.90 (10.00)	Liu et al. (2024) (14)
Female (%)		**Physiological parameter**	46.70	47.60	Liu et al. (2024) (14)
Duration of T2D (years)		8.56 (5.68)	9.06 (6.00)	8.81 (5.84)	Liu et al. (2024) (14)
Smoker (%)		27.50	27.50	27.50	Wang et al. (2017) (33)
SBP (mmHg)		131.89	131.89	131.89	Ji et al. (2013) (34)
LDL (mg/dL)		112.92	112.92	112.92	Ji et al. (2013) (34)
Height (m)		1.64	1.64	1.64	Gov.cn (35)
BMI (kg/m^2^)		25.92 (3.47)	25.42 (3.25)	25.67 (3.36)	Liu et al. (2024) (14)
History of complications, percentage					
Stroke		0.0600	0.0600	0.0600	Ji et al. (2015) (36)
CHF		0.0160	0.0160	0.0160	Quan et al. (2021) (37)
MI		0.0150	0.0150	0.0150	Quan et al. (2021) (37)
Angina		0.0172	0.0172	0.0172	WU et al. (2016) (37)
Revascularisation		0.0040	0.0040	0.0040	Quan et al. (2021) (37)
Neuropathy		0.2100	0.1990	0.2045	Liu et al. (2024) (14)
Blind		0.0000	0.0000	0.0000	WU et al. (2016) (16)
ESRD		0.0070	0.0070	0.0070	Quan et al. (2021) (16)
Treatment effects of various lines of therapy					
HbA1c (%)	Baseline	8.58 (0.93)	8.53 (0.90)	8.56 (0.92)	Liu et al. (2024) (14)
	Change	−1.88 (0.05)	−1.68 (0.05)		Liu et al. (2024) (14)
Weight (kg)	Baseline	70.72 (13.36)	69.02 (12.53)	69.87 (12.95)	Liu et al. (2024) (14)
	Change	−0.30 (0.29)	1.19 (0.29)		Liu et al. (2024) (14)
Any hypoglycaemia		35.20	40.50		Liu et al. (2024) (14)
Level 1 hypoglycaemia		33.10	39.50		Liu et al. (2024) (14)
Level 2 hypoglycaemia		6.90	8.60		Liu et al. (2024) (14)
Level 3 hypoglycaemia		0.00	0.00		Liu et al. (2024) (14)

The value in parentheses is SD. Given the limitations of the clinical data from the Soli-D trial, some model parameters were derived from other studies based on Chinese populations. The related limitations have been detailed in the Discussion section. HbA1c, Glycated hemoglobin A1c; SBP, Systolic blood pressure; Gov.cn, The Central People's Government of the People's Republic of China; CHF, Congestive heart failure; MI, Myocardial infarction; ESRD, End-stage renal disease; iGlarLixi, Insulin glargine and lixisenatide; IDegAsp, Insulin degludec and insulin aspart.

### 2.3 Inclusion and exclusion criteria

#### 2.3.1 Inclusion criteria:

- Adults aged ≥18 years with a diagnosis of T2D for at least 1 year.
- HbA1c at screening:

- 7.5%−11.0% for participants previously treated with metformin alone or metformin plus an SGLT-2i.
- 7.0%−10.0% for participants previously treated with metformin plus a second OAD (excluding SGLT-2i).

- BMI < 40 kg/m^2^ at screening.
- Treated with a stable dose of metformin (≥1000 mg or maximum tolerated dose), either alone or in combination with a second OAD (sulfonylureas, glinides, alpha-glucosidase inhibitors, DPP-4i, or SGLT-2i).
- Willingness to discontinue OADs other than metformin and SGLT-2i prior to randomization.

#### 2.3.2 Exclusion criteria:

- Previous insulin treatment within 1 year prior to screening.
- Previous use of glucose-lowering agents not included in the inclusion criteria.
- Use of weight-loss drugs within 3 months prior to screening.
- Discontinuation of previous treatment with a GLP-1 RA.

### 2.4 Cost and utility inputs

This study conducted a cost-effectiveness analysis from the perspective of the healthcare system. The cost incorporates diabetes management, treatment, and complications. Comprehensive cost data are presented in Table 2. Costs associated with routine diabetes management and diabetes-related complications were obtained from previous studies (16, 17). Medications-related costs primarily include insulin and injection supplies. Because both regimens involved once-daily insulin administration, needle costs were assumed to have minimal and comparable influence on overall outcomes; therefore, only the costs of the insulin formulations were included. Drug prices were sourced from Yaozh.com on Sep. 28, 2024 (18) and were based on the median of the lowest unit prices from provincial centralized procurement winning bids. Specifically, the cost per pen was $31.82 for iGlarLixi (3 ml: 0.3 mg) and $22.47 for iGlarLixi (3 ml: 0.15 mg). The cost per pen of IDegAsp (3 ml: 300 units) was $9.04. Drug costs per kilogram of body weight were calculated using data from the Soli-D clinical trial [1. All costs were adjusted to 2024 values using China's Consumer Price Index ^24^ and converted to US $ at an average 2024 exchange rate of $1 = 7.12 CNY (19).

**Table 2:** Cost parameters input for the BRAVO. The cost incorporates diabetes management, treatment, and complications. All costs are expressed in USD. Sources of cost data are indicated in the table.

Medication dosage and acquisition cost					
Parameters	iGlarLixi		IDegAsp		Source
Dose	iGlar: 0.40 U/kg; Lixi: 16.2 ug		0.48 U/kg		Liu et al. (2024) (14)
Drug acquisition costs ($)	0.0311		0.0145		Yao. (18)
Model inputs for complication treatment and disease management costs					
Parameters	Costs ($)	Source	Parameters	Costs ($)	Source
Disease management cost	1,349	WU et al. (2016) (16)	Revascularization surgery in the 1st year	3,370	Duan et al. (2019) (17)
MI			Revascularization surgery in the 2nd year and beyond	529	Duan et al. (2019) (17)
MI in the 1st year	11,573	Duan et al. (2019) (17)	ESRD		
MI in the 2nd year and beyond	3,659	Duan et al. (2019) (17)	ESRD in the 1st year	22,739	Duan et al. (2019) (17)
Angina			ESRD in the 2nd year and beyond	18,288	Duan et al. (2019) (17)
Angina in the 1st year	5,594	Duan et al. (2019) (17)	Acute complication		
Angina in the 2nd year and beyond	1,583	Duan et al. (2019) (17)	Severe Hypoglycemia	1,979	Duan et al. (2019) (17)
CHF			Non-severe hypoglycemic	126	Duan et al. (2019) (17)
CHF in the 1st year	5,545	Duan et al. (2019) (17)	Blindness		
CHF in the 2nd year and beyond	2,941	Duan et al. (2019) (17)	Blindness in the 1st year	334	WU et al. (2016) (16)
Stroke			Blindness in the 2nd year and beyond	110	WU et al. (2016) (16)
Stroke in the 1st year	4,583	Duan et al. (2019) (17)	SPSL		
Stroke in the 2nd year and beyond	2,267	Duan et al. (2019) (17)	SPSL in the 1st year	2,694	WU et al. (2016) (16)
Revascularization surgery			SPSL in the 2nd year and beyond	1,034	WU et al. (2016) (16)

iGlarLixi, Insulin glargine and lixisenatide; IDegAsp, Insulin degludec and insulin aspart; Yao., www.yaozh.com; MI, Myocardial infarction; CHF, Congestive heart failure; ESRD, End-stage renal disease; SPSL, Severe pressure sensation loss.

Baseline utility values and diabetes-related dis-utilities for individuals with type 2 diabetes were derived from health utility studies conducted in Chinese or broader Asian populations (Table 3).

**Table 3:** Utility values for each health state applied in the analysis. Health-related quality of life (utility) values and utility decline for each disease condition used in the BRAVO model. Sources of data are indicated in the table.

Health status/event	Utility	Utility decline	Source
Uncomplicated type 2 diabetes mellitus	0.881		Mok et al. (2021) (38)
MI	0.874	−0.007	Mok et al. (2021) (38)
Angina	0.864	−0.017	Mok et al. (2021) (38)
CHF	0.831	−0.050	Mok et al. (2021) (38)
Stroke	0.717	−0.164	Beaudet et al. (2014) (39)
Revascularization surgery	0.864	−0.017	Beaudet et al. (2014) (39)
ESRD	0.828	−0.053	Beaudet et al (2014) (39)
Severe vision loss/blindness	0.780	−0.101	Mok et al. (2021) (38)
SPSL	0.829	−0.052	Mok et al. (2021) (38)
Non-severe daytime/nocturnal hypoglycemia	0.876	−0.014	Beaudet et al. (2014) (39)
Severe hypoglycemia does not require medical assistanc	0.863	−0.0183	Mok et al. (2021) (38)
Requires medical assistance for severe day/night hypoglycemia	0.834	−0.047	Beaudet et al. (2014) (39)

MI, Myocardial infarction; CHF, Congestive heart failure; ESRD, End-stage renal disease; SPSL, Severe pressure sensation loss.

### 2.5 Time horizons

Given the average life expectancy of 78.2 years in China (National Health Commission, 2022) (20) and the mean baseline age of 56.9 years in the trial population, a 20-year time horizon was selected to model long-term disease progression. The model employed annual cycles to capture changes in health status from treatment initiation to either death or the end of the simulation period.

### 2.6 Analysis

Final outcomes assessed in this study included life years (LYs), quality-adjusted life years (QALYs), and total direct medical costs. The incremental cost-effectiveness ratio (ICER) for iGlarLixi compared to IDegAsp was calculated as the ratio of incremental cost to incremental QALYs gained.

The analysis incorporated an annual prediction of outcome incidences based on updated individual-level parameters from the preceding year. Key modeled outcomes comprised macrovascular and microvascular complications, along with yearly updates to individual clinical parameters. The primary outcomes of interest were macrovascular events, microvascular events, and all-cause mortality. Macrovascular complications included myocardial infarction (MI), stroke, congestive heart failure (CHF), and major adverse cardiovascular events (MACE), which encompass nonfatal MI, nonfatal stroke, and cardiovascular death. The composite microvascular outcome included end-stage renal disease (ESRD), blindness, and severe loss of pressure sensation (21). The willingness-to-pay (WTP) threshold was defined as three times China's per capita gross domestic product in 2024 ($13,448) (22), yielding a threshold of $40,344 per QALY.

Sensitivity and scenario analyses were performed to test robustness. One-way sensitivity analyses evaluated the impact of varying key input parameters, including costs associated with complications, drug prices, and general diabetes management expenses. Probabilistic sensitivity analysis (PSA) was conducted using 1,000 Monte Carlo simulations to explore parameter uncertainty across treatment efficacy, costs, utility values, and complication risks. All relevant models input—including utilities, risk estimates, and costs—were assumed to follow a normal distribution.

Scenario analyses were performed to assess the effect of alternative modeling assumptions on outcomes. First, the model was run using time horizons of 10, 20, and 30 years to examine the influence of simulation duration. Second, treatment effect attenuation was modeled by incorporating a decline in treatment efficacy after either 1 or 3 years, with subsequent disease progression aligned with the BRAVO model trajectory.

## 3 Results

### 3.1 Base-case and scenario analyses

Based on the 20-year simulation using the BRAVO model, individuals treated with iGlarLixi achieved 9.43 QALYs and 17.35 LYs, compared with 9.41 QALYs and 17.33 LYs in the IDegAsp group. This corresponds to an incremental gain of 0.03 QALYs and 0.01 LYs in favor of iGlarLixi. From a cost perspective, the estimated total medical expenditure for the iGlarLixi regimen was $34,436.38, compared with $34,737.76 for IDegAsp, resulting in a cost saving of $301.38. The corresponding ICER indicated that iGlarLixi was dominant—providing better outcomes at a lower cost—saving $11,907.09 per QALY gained compared with IDegAsp. In all tested scenarios, the ICER for IGlarLixi remained a dominant treatment over IDegAsp, supporting the robustness of the base-case findings (Table 4).

**Table 4:** Baseline analysis and scenario analysis results. Outcomes of the cost-effectiveness analyses under the base case and alternative scenarios. Results are presented as total costs, QALYs, ICERs, and other relevant indicators.

Analysis scenario	Outcome measures	iGlarLixi	IDegAsp	Incremental difference
Baseline analysis	Total cost, $	34,436.38	34,737.76	−301.38
	QALYs	9.43	9.41	0.0253
	Life-years	17.35	17.33	0.0236
	ICER	Dominant		−11,907.09
	NMB, $			641.61
Scenario analyses 1	Total cost, $	17,560.64	17,712.72	−152.08
	QALYs	6.30	6.29	0.0129
	Life-years	9.49	9.49	0.0039
	ICER	Dominant		−11,749.34
	NMB, $			325.56
Scenario analyses 2	Total cost, $	44,596.49	44,894.59	−298.10
	QALYs	10.59	10.55	0.0364
	Life-years	22.12	22.06	0.0644
	ICER	Dominant		−8,186.57
	NMB, $			787.61
Scenario analyses 3	Total cost, $	35,019.63	35,202.67	−183.04
	QALYs	9.39	9.36	0.0247
	Life-years	17.29	17.27	0.0250
	ICER	Dominant		−7400.95
	NMB, $			515.21
Scenario analyses 4	Total cost, $	34,695.75	34,932.23	−236.48
	QALYs	9.41	9.39	0.0266
	Life-years	17.33	17.30	0.0282
	ICER	Dominant		−8897.74
	NMB, $			594.20

Scenario analyses 1: simulation run for 10 years; Scenario analyses 2: simulation run for 30 years; Scenario analyses 3: treatment effects begin to diminish after 1 year; Scenario analyses 4: treatment effects begin to diminish after 2 year. iGlarLixi, Insulin glargine and lixisenatide; IDegAsp, Insulin degludec and insulin aspart; QALYs, Quality-adjusted life years; ICER, Incremental cost-effectiveness ratio; NMB, Net monetary benefit.

**Figure 1:**
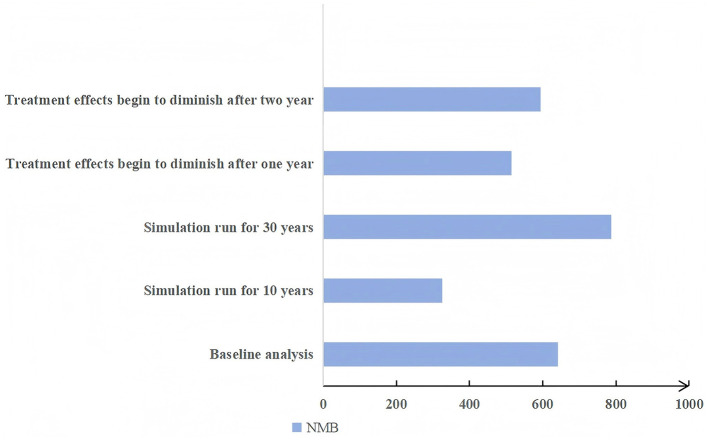
Net monetary benefit (NMB) of all scenario analyses comparing iGlarLixi and IDegAsp in Chinese adults with T2D inadequately controlled on oral antidiabetic drugs. Health and economic outcomes under different scenarios were simulated using the BRAVO model, with each bar representing the NMB of a given scenario.

Figure 1 shows the scenario analyses for net monetary benefit (NMB). In every scenario, NMB was positive, indicating that iGlarLixi is cost-effective compared with IDegAsp within the defined WTP threshold. Notably, the 30-year simulation scenario yielded the highest NMB, suggesting that the economic advantage of iGlarLixi becomes more pronounced over longer treatment durations.

Table 5 summarizes the cumulative incidence of diabetes-related complications across the two treatment groups. Individuals in the iGlarLixi group consistently exhibited lower complication rates than those in the IDegAsp group, with relative risk values below 1.0, indicating a reduced overall risk of diabetes-related complications.

**Table 5:** Cumulative incidence of complications over a lifetime time horizon. Modeled lifetime cumulative incidence of diabetes-related complications for iGlarLixi and IDegAsp treatment strategies.

Risk events	IGlarLixi (%)	IDegAsp (%)	Relative Risk (%)
Stroke	10.60	10.77	98.49
Non-fatal	9.65	9.80	98.45
Fatal	0.95	0.96	98.87
MI	9.97	10.04	99.34
Non-fatal	8.98	9.03	99.34
Fatal	1.00	1.00	99.34
CHF	10.35	10.67	97.08
Non-fatal	8.56	8.77	97.62
Fatal	1.80	1.90	94.61
Angina	5.10	5.25	97.12
Revascularization	11.35	11.65	97.44
ESRD	11.70	11.80	99.18
Blind	31.81	32.06	99.22
SPSL	26.27	26.63	98.66
All cause mortality	32.91	33.23	99.05
CVD mortality	16.93	17.40	97.28
MACE component	33.78	34.43	98.11

MI, Myocardial infarction; CHF, Congestive heart failure; ESRD, End-stage renal disease; SPSL, Severe pressure sensation loss; CVD, Cardiovascular disease; MACE, Major adverse cardiovascular event; iGlarLixi, Insulin glargine and lixisenatide; IDegAsp, Insulin degludec and insulin aspart.

### 3.2 One-way and probabilistic sensitivity analyses

**Figure 2:**
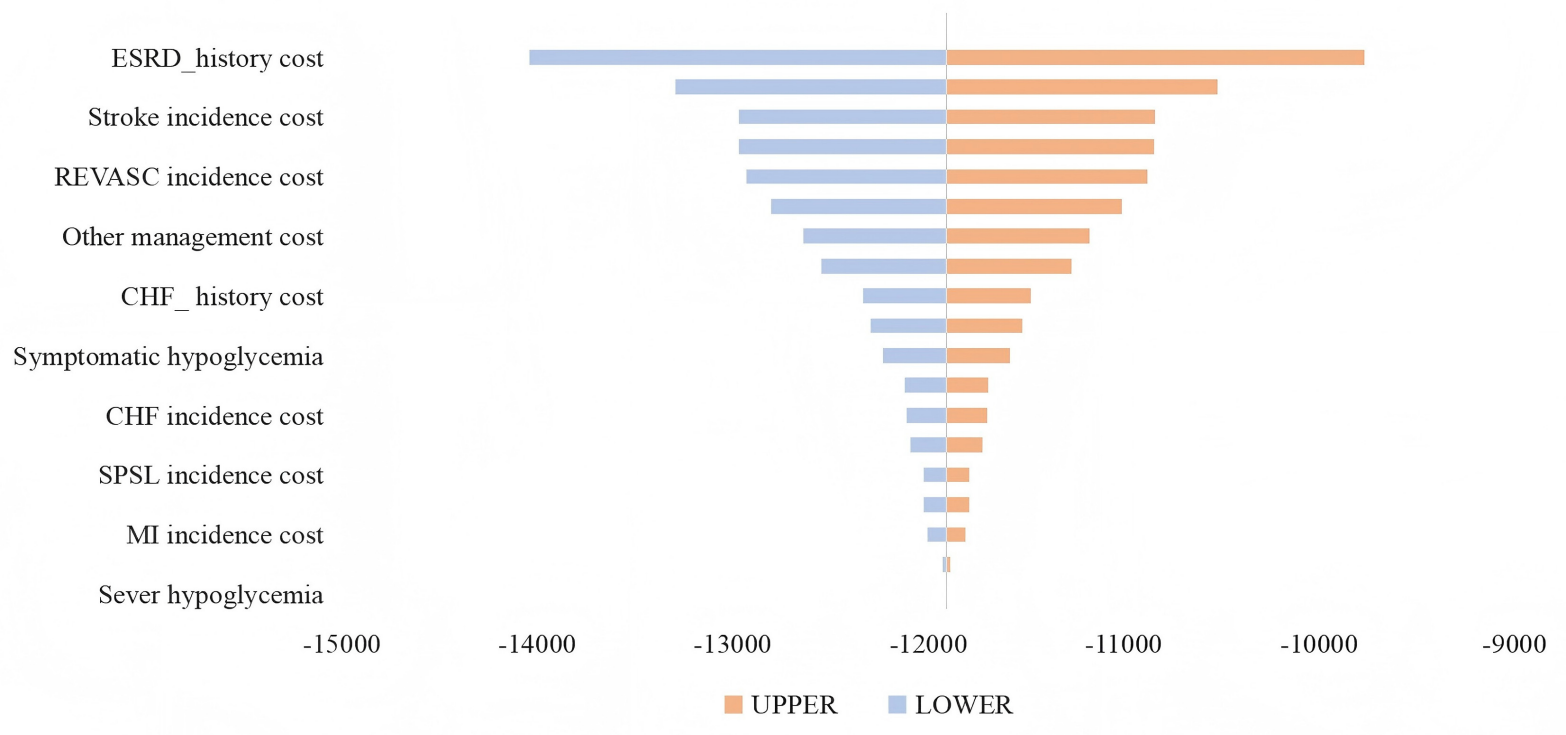
One-way sensitivity analysis tornado diagram comparing iGlarLixi and IDegAsp. The figure displays the influence of individual parameters on the ICER, with bars representing the range of ICER variation when each parameter is varied within its predefined limits.

**Figure 3:**
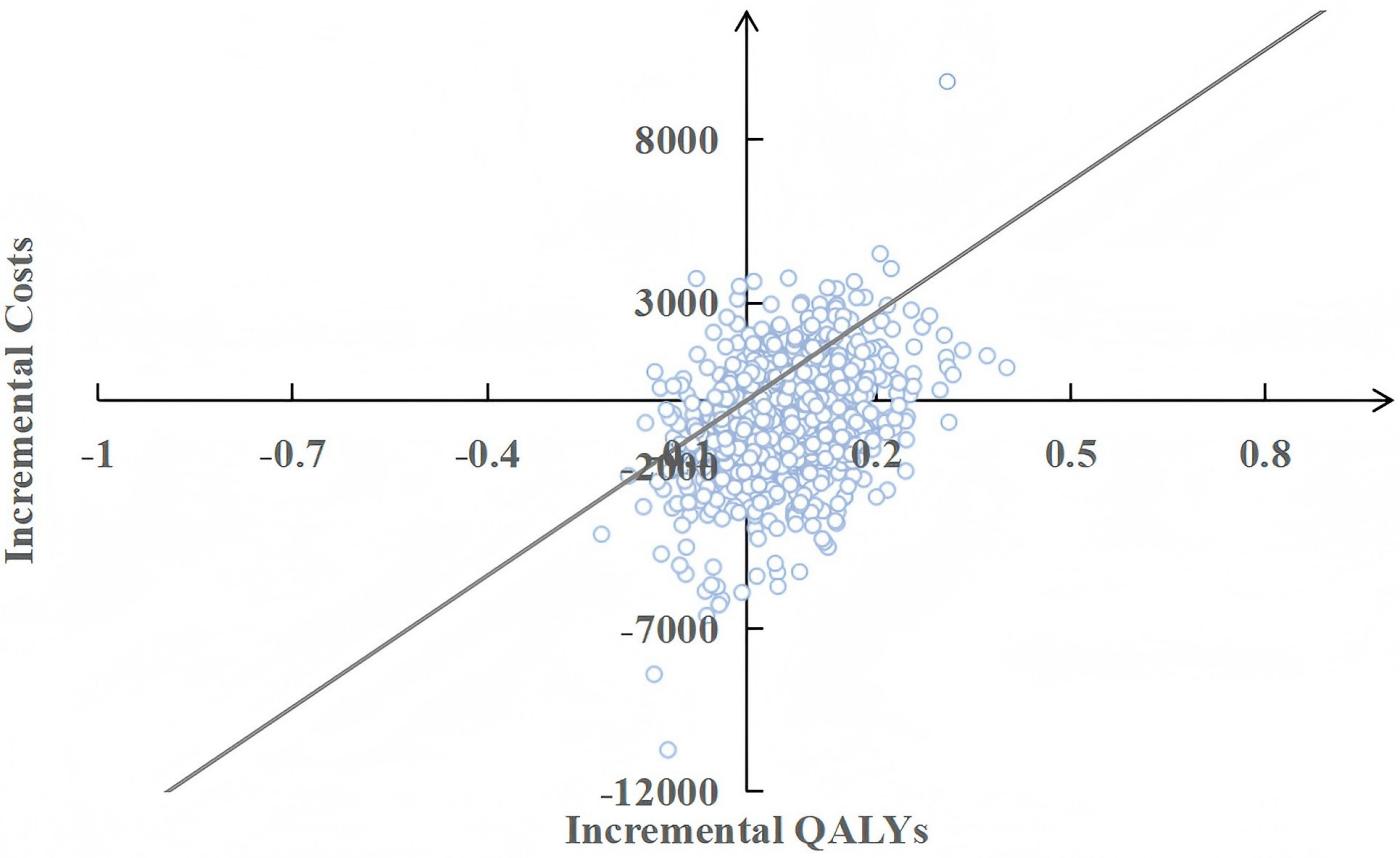
PSA scatterplot comparing iGlarLixi and IDegAsp. Each point represents an ICER estimate derived from a Monte Carlo simulation, illustrating the joint uncertainty in costs and effectiveness across 10 000 iterations.

**Figure 4:**
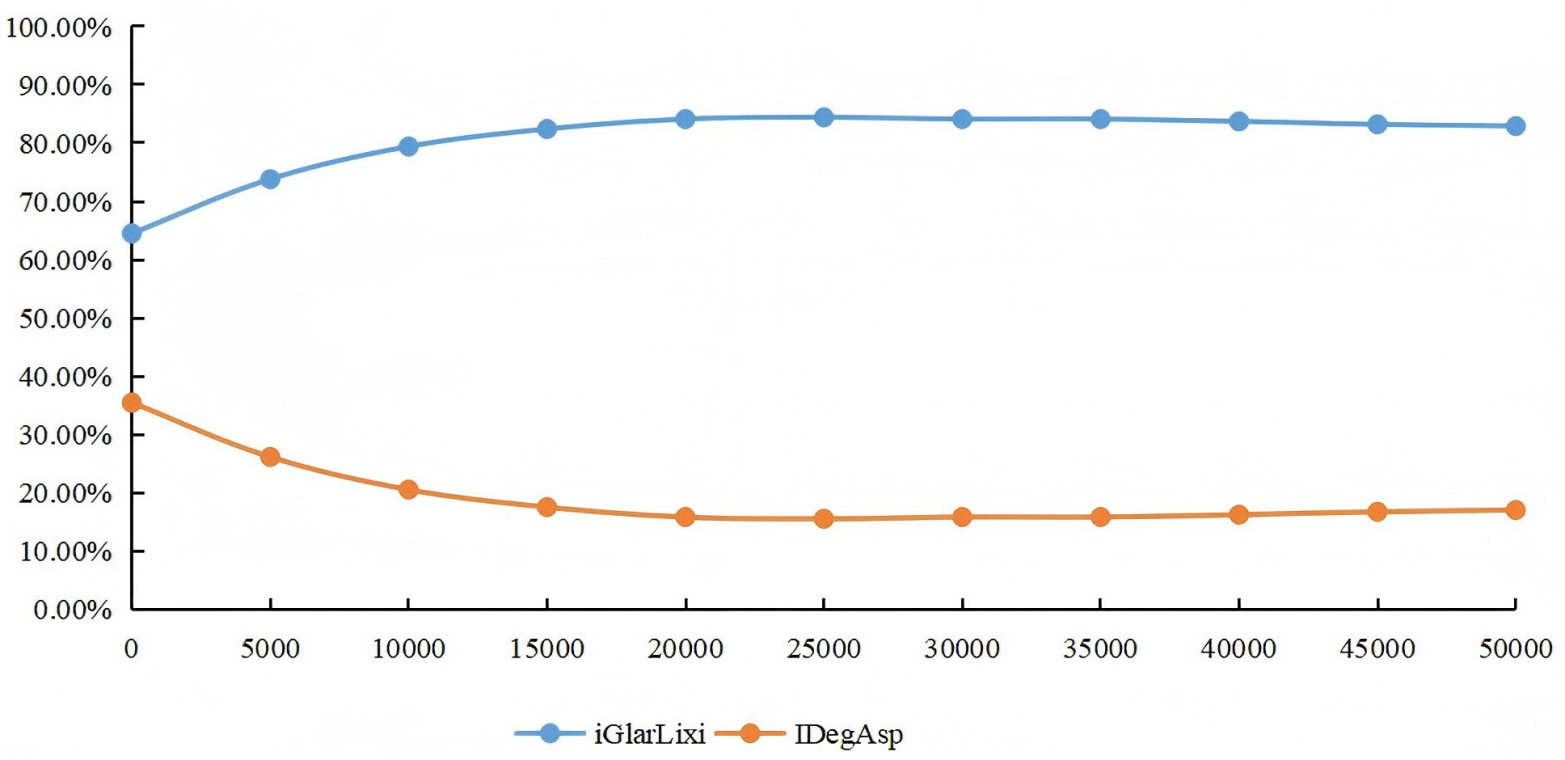
Cost-effectiveness acceptability curve (CEAC) comparing iGlarLixi and IDegAsp. The curve shows the probability of each treatment being cost-effective across a range of WTP thresholds, based on probabilistic sensitivity analysis results.

One-way sensitivity analysis showed that the costs associated with ESRD, MI, and stroke had the greatest impact on the model outcomes, with ESRD history cost having the largest effect (Figure 2). Figure 3 presents the scatter plot from the probabilistic sensitivity analysis (PSA) on the cost-effectiveness plane. The PSA points are distributed across all four quadrants, illustrating the variability in both incremental costs and incremental QALYs. At a willingness-to-pay (WTP) threshold of $40,344 per QALY, the probability that iGlarLixi is cost-effective compared with IDegAsp was 83%, while the probability of achieving a positive incremental QALY was 78.6%. These findings highlight the robustness of the model results and the potential economic and clinical benefits of iGlarLixi. Figure 4 shows the cost-effectiveness acceptability curve (CEAC), which demonstrates that the probability of iGlarLixi being cost-effective increases steadily with rising WTP thresholds before reaching a plateau.

## 4 Discussion

To the best of our knowledge, this is the first study to evaluate the long-term cost-effectiveness of iGlarLixi compared to IDegAsp based on the Phase 3 randomized controlled trial (RCT) data. Using multiple reasonable time horizon simulations and varying durations of treatment effects, our findings demonstrate that iGlarLixi is a cost-effective strategy in individuals with poorly controlled type 2 diabetes. Based on the Soli-D trial (14), the BRAVO model simulations project that iGlarLixi superior to IDegAsp in reducing the long-term risk of diabetes-related macrovascular and microvascular complications, as well as all-cause mortality.

This study provides valuable insights into the clinical applications and policy decisions. The latest clinical consensus has established that combining BI with GLP-1 RAs offers superior glycemic control compared to either agent alone, while also yielding less weight gain and lower hypoglycemia risk than intensified insulin regimens, and improved gastrointestinal tolerability relative to GLP-1 RA monotherapy (23). iGlarLixi has demonstrated significant reductions in HbA1c and mitigated insulin-associated weight gain without increasing hypoglycemia risk in patients with inadequately controlled HbA1c (24). Liu L. et al. reported that 49.3% of patients receiving GLP-1 RAs discontinued therapy after 1 year, indicating that adherence remains suboptimal (25). In contrast, iGlarLixi delivers two complementary glucose-lowering mechanisms within a simplified regimen, thereby reducing both the practical burden and psychological barriers associated with treatment, and potentially improving patient adherence (26). These clinical benefits highlight the potential value of iGlarLixi, which is further reinforced by recent policy changes in China that enhance patient access to innovative therapies.

Against the backdrop of the National Healthcare Security Administration's ongoing efforts to enhance the refined management of the MI fund and implement policy-driven drug price negotiations, the inclusion of fixed-ratio insulin combination therapies in China's national reimbursement drug list has significantly reduced patients' out-of-pocket expenses and expanded access to advanced diabetes treatments. According to the 2024 National Statistical Bulletin on the Development of Healthcare Security (27), the average reimbursement rate for covered inpatient expenses under the resident MI scheme reached 68.6% in 2024—an increase of 0.5 percentage points from 2023—with particularly notable improvements in reimbursement for chronic disease medications. In the same year, MI negotiation led to an average price reduction of 63%, including 15 newly added agents for chronic diseases such as diabetes. These changes have substantially enhanced both affordability and medication adherence, particularly in primary care settings. As reported in the China Guidelines for the Prevention and Treatment of T2D (2024) (28), over 90% of individuals with diabetes in China are diagnosed with T2D. Under the expanded MI coverage, this population has gained greater access to newer, more efficacious combination therapies, such as iGlarLixi, which may reduce the risk of complications associated with inadequate glycemic control. Beyond improving accessibility, the wider adoption of iGlarLixi has important implications for healthcare resource utilization and long-term sustainability of the MI fund. From the perspective of MI fund expenditure, diabetes-related macrovascular and renal complications—such as ESRD, myocardial infarction (MI was used for medical insurance before), and stroke—are associated with prolonged, high-cost treatment courses and intensive healthcare intervention. Once the disease progresses to advanced stages, therapies such as dialysis, cardiac procedures, or neurorehabilitation become not only financially burdensome but also markedly imparir patients' quality of life. By reducing the incidence of such complications, iGlarLixi may contribute to delaying disease progression at the individual level while alleviating treatment burdens at the institutions level, particularly across secondary and tertiary facilities. Ultimately, these benefits may help contain the growth of per capita healthcare expenditures and support the long-term sustainability of China's MI fund.

Our findings are consistent with those of previously published studies. Although no direct cost-effectiveness analyses comparing iGlarLixi with IDegAsp have been conducted in other countries, existing research evaluating iGlarLixi against alternative BI regimens has demonstrated favorable economic outcomes. For example, McCrimmon et al. (29) assessed the cost-effectiveness of iGlarLixi vs. biphasic insulin aspart 30 in individuals with T2D inadequately controlled on BI, reporting an ICER of £13,598 per QALY. In a separate study, McCrimmon et al. (30) compared iGlarLixi with iDegLira among individuals whose glycemic control was inadequate despite GLP-1 RA therapy and found that iGlarLixi led to a cost saving of £715,333.33 per QALY. To date, only one study has directly evaluated the cost-effectiveness of iGlarlixi and IDegAsp. That analysis, conducted prior to iGlarLixi's launch in China, reported an ICER of $443.7 per QALY gained based on projected pricing assumptions (31), and concluded that iGlarLixi was a cost-effective alternative. Collectively, these findings underscore the strong cost-effectiveness profile of iGlarLixi across a range of comparator therapies, strengthening the evidence base for informed clinical and policy decision-making.

One-way sensitivity analysis identified the costs associated with ESRD, MI, and stroke as the most influential parameters affecting the model outcomes. Scenario analyses further confirmed the robustness of the results, with all ICER values remaining below the WTP threshold across all tested scenarios. PSA demonstrated that treatment with iGlarLixi had an approximately 83% probability of being cost-effective compared to IDegAsp. In addition to economic outcomes, model simulations predicted a lower cumulative incidence of diabetes-related complications in the iGlarLixi group than in the IDegAsp group, suggesting that iGlarLixi offers advantages in both cost-effectiveness and clinical efficacy. These findings are consistent with those of Shao et al. (13), who used the BRAVO model based on data from the LixiLan-O and ACCORD trials to simulate the 5-year risk of diabetes-related complications. Their study showed that long-term use of iGlarLixi may substantially reduce the risk of complications in individuals with T2D at elevated cardiovascular risk. The convergence of evidence from multiple analyses reinforces the robustness and external validity of our findings. Taken together, these results suggest that iGlarLixi demonstrates clear superiority over IDegAsp in terms of both economic value and clinical benefit, supporting its broader adoption within healthcare systems aiming to optimize outcomes and resource allocation for patients with T2D.

Notably, the BRAVO model offers a distinct methodological advantage in its ability to capture the additional risks associated with hypoglycemia-related complications and mortality (32). These strengths make BRAVO particularly well-suited for evaluating the long-term clinical and economic outcomes of interventions such as iGlarLixi. Furthermore, this analysis incorporated the reimbursed price of iGlarLixi following its inclusion in NRDL, thereby enhancing the real-world relevance of the cost estimates within the context of the Chinese healthcare payment system. In addition, efficacy and safety data were derived from a RCT conducted specifically in a Chinese population, enabling the model to more accurately reflect local patient characteristics and treatment responses.

Despite its strengths, this study has several limitations. First, the analysis is based on relatively short-term clinical trial data (27 weeks) to extrapolate long-term health and economic outcomes—a common limitation in cost-effectiveness analyses. However, the robustness of our findings was supported by extensive sensitivity analyses across a wide range of key parameter assumptions, helping to mitigate uncertainties associated with the limited trial duration. Second, the use of peripheral vascular lesion data as a surrogate for revascularization surgery, and neurological lesion data as a proxy for severe loss of pressure sensation, may have introduced some degree of bias in estimating clinical outcomes. These approximations, while necessary due to data availability, underscore the need for more granular clinical inputs in future modeling efforts. Third, the time elapsed since the ACCORD trial—on which the BRAVO model's risk equations are based—may limit their relevance to contemporary clinical practice. Fourth, although the model was parameterized using Chinese and broader Asian data where available, it was originally developed based on baseline characteristics from a U.S. population, which could affect the accuracy of outcome estimates when applied to China. Further localized studies are warranted to calibrate and validate the model for use in Chinese healthcare settings. Fifth, medication adherence, a well-recognized determinant of glycemic control and long-term outcomes, was not considered because adherence data were not collected in the source clinical trial. Consequently, the impact of adherence on long-term cost-effectiveness remains unquantified. Future studies incorporating real-world adherence data would help address this limitation. Sixth, this study was conducted from the perspective of the Chinese healthcare system, and the cost data and treatment patterns reflected local clinical practice; therefore, the findings are context-specific. When extrapolating these results to other countries or regions, caution is needed, as differences in drug pricing, reimbursement policies, and treatment patterns may influence cost-effectiveness outcomes. Future studies are needed to calibrate the model using local data and further assess its applicability in different healthcare settings.

## 5 Conclusions

From the perspective of the Chinese healthcare system, compared to IDegAsp, iGlarLixi demonstrates superior cost-effectiveness in individuals with type 2 diabetes inadequately controlled with oral antidiabetic therapy.

## Data Availability

The original contributions presented in the study are included in the article/supplementary material, further inquiries can be directed to the corresponding author.
